# Dissection of the Activity of Agricultural Fungicides against Clinical *Aspergillus* Isolates with and without Environmentally and Medically Induced Azole Resistance

**DOI:** 10.3390/jof7030205

**Published:** 2021-03-11

**Authors:** Karin Meinike Jørgensen, Marie Helleberg, Rasmus Krøger Hare, Lise Nistrup Jørgensen, Maiken Cavling Arendrup

**Affiliations:** 1 Unit for Mycology, Statens Serum Institut, 2300 Copenhagen, Denmark; kmj@ssi.dk (K.M.J.); rmj@ssi.dk (R.K.H.); 2 Department of Infectious Diseases, Rigshospitalet, 2100 Copenhagen, Denmark; Marie.Helleberg@regionh.dk; 3 Department of Agroecology—Crop Health, Aarhus University-Flakkebjerg, 4200 Slagelse, Denmark; lisen.jorgensen@agro.au.dk; 4 Department of Clinical Medicine, Copenhagen University, 2100 Copenhagen, Denmark; 5 Department of Clinical Microbiology, Rigshospitalet, 2100 Copenhagen, Denmark

**Keywords:** *Aspergillus*, *A. fumigatus*, *A. terreus*, *A. flavus*, resistance, fungicide, azole, environmental resistance

## Abstract

Azole resistance is an emerging problem in patients with aspergillosis. The role of fungicides for resistance development and occurrence is not fully elucidated. EUCAST reference MICs of 17 fungicides (11 azoles and 6 others), five azole fungicide metabolites and four medical triazoles were examined against two reference and 28 clinical isolates of *A. fumigatus*, *A. flavus* and *A. terreus* with (*n* = 12) and without (*n* = 16) resistance mutations. Eight/11 azole fungicides were active against wild-type *A. fumigatus*, *A. flavus* and *A. terreus*, including four (metconazole, prothioconazole-desthio, prochloraz and imazalil) with low MIC_50_ (≤2 mg/L) against all three species and epoxiconazole, propiconazole, tebuconazole and difenoconazole also against wild-type *A. terreus*. Mefentrifluconazole, azole metabolites and non-azole fungicides MICs were >16 mg/L against *A. fumigatus* although partial growth inhibition was found with mefentrifluconazole. Moreover, mefentrifluconazole and axozystrobin were active against wild-type *A. terreus*. Increased MICs (≥3 dilutions) were found for TR_34_/L98H, TR_34_^(3)^/L98H, TR_46_/Y121F/T289A and G432S compared to wild-type *A. fumigatus* for epoxiconazole, propiconazole, tebuconazole, difenoconazole, prochloraz, imazalil and metconazole (except G432S), and for prothioconazole-desthio against TR_46_/Y121F/T289A, specifically. Increased MICs were found in *A. fumigatus* harbouring G54R, M220K and M220R alterations for five, one and one azole fungicides, respectively, compared to MICs against wild-type *A. fumigatus*. Similarly, increased MICs wer found for *A. terreus* with G51A, M217I and Y491H alterations for five, six and two azole fungicides, respectively. Azole fungicides showed activity against wild-type *A. fumigatus*, *A. terreus* and *A. flavus*, but not against all mutant isolates, suggesting the environmental route of azole resistance may have a role for all three species.

## 1. Introduction

*Aspergillus fumigatus*, *Aspergillus flavus* and *Aspergillus terreus* exist ubiquitously in the environment. However, they are also important human pathogens causing allergic bronchopulmonary aspergillosis in patients with asthma or cystic fibrosis, chronic pulmonary aspergillosis in those with impaired lung tissue architecture and severe invasive infections in immunocompromised patients [1,2,3]. *A. fumigatus* is the most common cause of human *Aspergillus* infections globally, whereas the incidence of the other two species varies geographically. *A. flavus* is the second most common species in Asia but *A. terreus* the second most common species in Austria [1,3]. *A. fumigatus* is known to be abundant in decaying vegetation in fields, forests, and compost heaps while both *A. terreus* and *A. flavus* are known to be abundant in soils [4,5]. *A. terreus* is found in compost heaps, grassland soils and soil from potted plants from where it has been identified as a source for a hospital outbreak [6]. This species has also been reported as a contaminant of plant products like stored corn, barley and peanuts [7]. In contrast to the two others, *A. flavus* is considered a plant pathogen as it destroys agricultural products (corn, legumes, nuts, etc.) mainly in tropical and subtropical regions [8]. It is furthermore known for its ability to produce aflatoxin that may cause hepatitis, cancer and death.

Voriconazole and isavuconazole are licensed as first line agents for invasive *Aspergillus* infections, while other azoles (posaconazole and itraconazole) are alternatives [9,10,11]. Echinocandins and polyenes can also be used to treat *Aspergillus* infections but are less efficacious and in addition, polyenes are associated with substantial toxicity and low activity against *A. terreus* and *A. flavus*. Currently, there are no approved oral alternatives to the triazole drugs [9,10].

Azole resistance in *Aspergillus* is associated with substantially increased mortality [11]. Moreover, it is a global problem and the incidence of invasive *Aspergillus* infections caused by azole resistant strains is increasing [2,12,13]. Azole resistant infections can arise in the individual patient during long-term therapy (the patient route) or be acquired due to inhalation of resistant *A. fumigatus* spores present in the environment (the environmental route) [14,15,16]. In agriculture, plant pathogenic fungi have a negative effect on crops and can lead to significant economic losses, which is why fungicides are widely used [17,18]. In addition, azoles are also used as growth regulators in both arable and flower-plant production. Several classes of fungicides are used in agriculture, including succinate dehydrogenase inhibitors SDHIs, phthalimide, QoIs/strobilurins and imidazole and triazole fungicides (DMIs). The latter includes agents that have molecule characteristics very similar to the triazoles used in medicine for treatment of human infections and which have been associated with induction of a tandem repeat mechanism in *A. fumigatus* in vitro [14]. The role of agricultural fungicides for development of azole resistance in *Aspergillus* in the environment and of azole resistant *Aspergillus* infections in humans is debated [16,17,19].

The most common azole resistance mechanism in *A. fumigatus* combines point mutation(s) in the coding sequence of the *cyp51A* gene and an insertion of a tandem repeat in the promoter region of this gene that leads to its overexpression (TR_34_/L98H and TR_46_/Y121F/T289A), and is presumed to be of environmental origin [14,20,21,22]. Resistance can also be induced by long-term triazole treatment and is usually caused by point mutations in the *cyp51A* gene of both *A. fumigatus* and *A. terreus* [2,15,23]. The mechanisms causing triazole resistance in *A. flavus* are less well characterised. However, recent studies have identified a number of azole resistant isolates and documented Cyp51A, Cyp51B or Cyp51C target enzyme alterations [24,25,26,27,28], upregulation of target gene expression [26,29], or efflux pump upregulation [26,29,30] in the resistant isolates. Moreover, resistance has been suggested among environmental *A. flavus* isolates in Vietnam [31] and Argentina [25].

A feature characteristic for antimicrobial agents involved in the development or selection of resistance is to possess a higher activity against wild-type isolates than against drug resistant mutants. We compared the MICs of agricultural fungicides and medical triazoles against *Aspergillus* spp. isolates with wild-type susceptibility and those with resistance mutations. Whereas previous studies have focused on fungicide MICs against *A. fumigatus* and most on TR_34_/L98H, this study investigated 17 fungicides, of which 11 were agricultural azoles, and four medical mould active triazoles, and five fungicide metabolites against 12 mutant, 16 wild-type and two QC strains of *A. fumigatus*, *A. terreus* and *A. flavus*.

## 2. Materials and Methods

### 2.1. Isolates

Twenty-eight clinical isolates and two reference strains (*A. fumigatus* ATCC 204305 and *A. flavus* ATCC 204304) were included. Of the 28 clinical isolates, 16 were *A. fumigatus* sensu stricto (seven wild-type and nine with the following resistance alterations: TR_34_/L98H, TR_34_^(3)^/L98H, TR_46_/Y121F/T289A, TR_120_/F46Y/M172V/E427K, G432S, G54A, G54R, M220K and M220R), 10 were *A. terreus* (seven wild-type and three with resistance alterations: G51A, M217I, Y491H) and two were *A. flavus* (both wild-type). Species identification was done by classical techniques, followed by thermotolerance (incubation at 50 °C) for discriminating *A. fumigatus* sensu stricto from cryptic species, and by β-tubulin and calmodulin sequencing of *A. terreus* and *A. flavus* isolates [32]. The calmodulin sequencing was performed using a slightly modified method based upon Hong et al. and the following primers: ASP-CMD_F: CCGAGTACAAGGAGGCCTTC and ASP-CMD_R: TTTYTGCATCATRAGYTGGAC [33]. The underlying target gene mutation of the included mutants had been characterized by PCR amplification and sequence analysis of the *cyp51A* gene [16,34].

### 2.2. Antifungals

Seventeen agricultural fungicides (test range 32–0.03 mg/L), five azole fungicide metabolites (test range 16–0.016 mg/L) and four medical azoles were included (test range 32–0.03 mg/L). The agricultural fungicides (bixafen, boscalid, fluxapyroxad, fluopyram, folpet, azoxystrobin, prothioconazole, paclobutrazole, epoxiconazole, propiconazole, tebuconazole, difenoconazole, metconazole, prothiozonacole-desthio, prochloraz, imazalil were purchased from Sigma-Aldrich, Søborg, Denmark, whereas mefentrifluconazol was purchased from LGC Standards (Teddington, Middlesex, United Kingdom www.lgcstandards.com, accessed on 26 January 2021). The metabolites included: 1,2,3-triazole (Sigma-Aldrich, Søborg, Denmark), 1,2,4-triazole and triazole sulfonamide (Toronto Research Chemicals, Toronto, Canada), and triazole alanine and triazole acetate (HPC Standards GmbH, Borsdorf, Germany). The medical antifungal agents used were itraconazole, voriconazole, isavuconazole and posaconazole (all from Sigma-Aldrich, Søborg, Denmark, except isavuconazole, which derived from Basilea).

### 2.3. Susceptibility Testing

The isolates were tested according to the EUCAST E.Def 9.3.2 microdilution method, with standard filtration (11-nm filter) of the inoculum [35]. Susceptibility testing was performed once, but repeated if growth curves were abnormal (bumpy) or growth was insufficient. Stock solutions of the antifungals were prepared at 5000 mg/L in dimethyl sulfoxide (Sigma-Aldrich, Søborg, Denmark). Cell culture-treated microtitre polystyrene plates (Nunc microwell 96-well microplates, catalog no. 167008; Thermo Fisher Scientific) were used throughout. At 48 h, the MIC was determined visually as the concentrations that produced a complete inhibition of growth. For mefentriazole specifically the plates were also read spectrophometrically and spectrophotometric MICs (spec-MICs) were determined using 50, 60, 70, 80 and 90% inhibition of the optical density as endpoints (Table S1). As breakpoints do not exist for fungicides against *Aspergillus*, we regarded isolates resistant when the MIC was >16 mg/L.

### 2.4. Data Analysis

The MIC_50,_ defined as the minimum concentration at which 50% of the isolates were inhibited was determined for the wild-type isolates of *A. fumigatus* and *A. terreus*. The relative efficacy of fungicides, fungicide metabolites and medical triazoles against wild-type versus mutant isolates was determined as the number of twofold dilution step differences in concentration that caused complete inhibition of growth of *Aspergillus* by determining the log2(mutant MIC) – log2(wild-type MIC_50_ ). For these calculations, MICs of >32 mg/L were translated to 64 mg/L. A log2 MIC difference of ≥3 (for example for MICs 8 vs. 1 mg/L) was considered significant.

## 3. Results

Seventeen agricultural fungicides, five fungicide metabolites and four medical triazoles, were tested against wild-type and mutant isolates of *A. fumigatus*, *A. terreus* and *A. flavus*. The susceptibility patterns of the medical azoles were in agreement with the well-known patterns confirming a reliable performance of the susceptibility testing and correct identification of species and underlying resistance mutations (Table 1) [2,16].

### 3.1. Fungicide Activity against Wild-Type Aspergillus Isolates

Nine azole fungicides displayed in vitro activity against wild-type *A. fumigatus* using a complete growth inhibition endpoint. Of these, four were highly active with MIC_50_ values of 0.06–0.5 mg/L (metconazole, prothioconazole-desthio, prochloraz and imazalil), and five were moderately active with MIC_50_ values of 2–16 mg/L (paclobutrazole, epoxiconazole, propiconazole, tebuconazole, and difenoconazole) (Table 1). Ten azole fungicides were in vitro active against *A. terreus*. These included five highly active difenoconazole, metconazole, prothioconazole-desthio, prochloraz and imazalil with MIC_50_ ≤ 1 mg/L and five that displayed weaker activity with MIC_50_ 2–8 mg/L (mefentrifluconazole, paclobutrazole, epoxiconazole, propiconazole, tebuconazole). Finally, nine of these were also active against wild-type isolates of *A. flavus*: metconazole, prothioconazole-desthio, prochloraz and imazalil with MIC_50_ values of 0.5–2 mg/L, and paclobutrazole, epoxiconazole, propiconazole, tebuconazole, and difenoconazole with MIC_50_ values of 8–16 mg/L. No activity was observed for the fungicide metabolites or the non-azole fungicides against wild-type isolates of the three *Aspergillus* species, with the exception azoxystrobin against *A. terreus* (MIC_50_ of 0.58 mg/L, Table 1).

### 3.2. Activity of Azole Fungicides against cyp51A Mutant Aspergillus

The four mutant *A. fumigatus* with tandem repeats in the promotor region of the *cyp51A* gene were resistant (MIC > 16 mg/L) to prothioconazole, mefentrifluconazole, paclobutrazole, epoxiconazole, propiconazole, tebuconazole and difenoconazole. Metconazole, prothioconazole-desthio, prochloraz and imazalil were active against TR_34_/L98H, TR_34_^(3)^/L98H, and TR_120_/F46Y/M172V/E427K (MICs of 0.125–8 mg/L) whereas metconazole (MIC 16 mg/L) and prothioconazole-desthio (1 mg/L) were the only agents with in vitro activity against TR_46_/Y121F/T289A. Among the isolates with single point mutations in the target gene, the susceptibility of the G432S mutant resembled that of the TR_34_/L98H, whereas the remaining mutants of *A. fumigatus* and *A. terreus* with single point mutations were more susceptible than the isolate harbouring the TR_34_/L98H to one or more of the following fungicides: epoxiconazole, propiconazole, tebuconazole, difenoconazole (Table 1).

### 3.3. The Relative Susceptibility of wt and Mutant Isolates

The relative susceptibility of wt and mutant isolates to azole fungicides was determined as the difference between log2 transformed MICs for mutants and wt isolates (Table 2). A log2 MIC difference of three or greater was found for the TR_34_/L98H, TR_34_^(3)^/L98H, TR_46_/Y121F/T289A and G432S mutants compared to wild-type *A. fumigatus* with epoxiconazole, propiconazole, tebuconazole, difenoconazole, metconazole (except G432S), prochloraz and imazalil. In addition, a log2 MIC difference of four was found for prothioconazole-desthio against TR_46_/Y121F/T289A but not TR_34_ tandem repeat mutants. The azole fungicides that displayed the greatest MIC elevation against the TR_34_/L98H and TR_34_^(3)^/L98H were difenoconazole and imazalil (four to five two-fold dilutions), whereas tebuconazole, difenoconazole, metconazole, prothioconazole-desthio, prochloraz and imazalil displayed the greatest MIC elevation against TR_46_/Y121F/T289A (four to seven two-fold dilutions).

Among the remaining mutants (isolated from patients with medical azole exposure), MIC elevations of three or more two-fold dilutions to several azole fungicides were found in *A. fumigatus* strains harbouring the TR_120_/F46Y/M172V/E427K (particularly difenoconazole) and G54R (particularly metconazole, prothioconazole-desthio and prochloraz) alterations, but not in isolates harbouring either the G54A, M220K (except epoxiconazole) or M220R (except prochloraz) alterations (Table 2). On the contrary, in *A. terreus*, MIC elevations of three or more dilutions were observed in isolates harbouring the G51A, and M217I alterations for epoxiconazole, propiconazole, tebuconazole, difenoconazole and mefentrifluconazole, and for the M217I specifically also for prochloraz. Finally, the susceptibility of the Y491H mutant *A. terreus* was less affected as a three-dilution elevation of MICs was only observed for two compounds (mefentrifluconazole and prochloraz).

### 3.4. Mefentriflucon Azole Displayed an Atypical Inhibition Pattern against Aspergillus

Complete growth inhibition was not achieved for mefentrifluconazole against neither wild-type nor mutant isolates of *A. fumigatus* and *A. flavus* even at the highest concentration tested (32 mg/L). However, partial inhibition was consistently observed for all three *Aspergillus* species at the higher concentrations and therefore endpoints were determined based on a range of 50% to 90% growth inhibition endpoints (Table S1). A ≥3 log2 MIC elevation (MIC ≥ 16 mg/L) compared to the wild-type (MIC_50_ 4 mg/L) was observed for *A. fumigatus* mutants harbouring TR_34_/L98H, TR_34_^(3)^/L98H, TR_46_/Y121F/T289A, TR_120_/F46Y/M172V/E427K and the M220R as well as for all three mutants of *A. terreus* (MICs 16->16 mg/L) compared to the wild-type (MIC_50_ 2 mg/L) when the spectrophotometric 50% inhibition endpoint was adopted.

### 3.5. Activity of Non-Azole Fungicides and Azole Fungicide Metabolites against Aspergillus

Whereas the non-azole fungicides were inactive against wild-type *A. fumigatus* and *A. flavus,* the SDHI fungicides bixafen, boscalid and fluxapyroxad (anilid) displayed activity against the *A. fumigatus* mutants TR_120_/F46Y/M172V/E427K, G432S and G54A (MICs 1–4 mg/L) (Table 1). Similarly, activity was observed of the SDHI fungicide fluopyram (a benzamide) against the TR_120_/F46Y/M172V/E427K and G54A *A. fumigatus* mutants (MICs 4 mg/L) and of azoxystrobin (QoI /strobilurin) against the TR_120_/F46Y/M172V/E427K mutant (MIC 8 mg/L). In contrast, only azoxystrobin displayed activity against wild-type isolates and the Y491H mutant of *A.* terreus, but not against the G54A and M217I mutants of *A. terreus*.

## 4. Discussion

The circumstantial evidence suggesting that TR_34_/L98H, TR_46_/Y121F/T289A and the less common TR_46_^(3)^/Y121F/T289A and TR_53_ azole resistance mechanisms in *A. fumigatus* originate from the environment is compelling [17,20,36,37]. This is in part because susceptible isogenic counterparts have never been isolated in humans and because resistant infections are diagnosed in azole naïve patients. However, it has become clear over the recent years that isolates with tandem repeats can also occasionally arise in humans during medical therapy. Thus, an isolate with a 120 base pair tandem repeat in the promotor region (TR_120_/F46Y/M172V/E427K) was recently demonstrated to have emerged in a patient during azole therapy [38]. It is also well documented that the list of point mutations in the *cyp51A* target gene that can arise during medical azole therapy and cause resistance is long and growing [17]. The single amino acid alterations M220K and M220R have only been found in azole-exposed patients. Nevertheless, it has become clear that several point mutations causing azole resistance can also be found in environmental isolates. For example, *A. fumigatus* isolates harbouring single point target gene mutations have been found in the environment including G54A in Germany [39], G54E in Italy, India, Romania, Tanzania, and Argentina [40,41,42,43], G54R in Switzerland and Thailand [44,45], M220I in Germany [39], P216L and H285Y in France [46,47], and G448S in China [48]. Moreover, clinical *A. flavus* isolates harbouring point mutations conferring Cyp51A P214L (itraconazole and posaconazole resistant) or Cyp51C H349R (pan human azole resistant) alterations displayed cross resistance to imazalil, prochloraz, metconazole, tebuconazole, epoxiconazole, and bromuconazole [28]. Besides an environmental itraconazole and voriconazole resistant *A. flavus* isolate from Argentina was found to harbour Cyp51C S361W and N423D alterations [25]. On this background, it is plausible that selection of azole resistance in the environment can take place not only in *A. fumigatus* but also in other clinically relevant *Aspergillus* species and that the underlying mechanisms are not limited to those consisting of the combination of tandem repeats and target gene mutations. Therefore, in this study we investigated the differential activity of fungicides (and azole fungicide metabolites) against wild-type and various mutant isolates of *A. fumigatus*, *A. flavus* and *A. terreus*. 

Epoxiconazole, propiconazole, tebuconazole and difenoconazole have previously been associated with selection of the TR_34_/L98H resistance mechanism in *A. fumigatus* [14]. We confirmed activity of these agents against wild-type *A. fumigatus* but also against wild-type *A. terreus* and *A. flavus* suggesting they may also pose a selection pressure for resistance in these species. An MIC elevation comparable to that seen in TR_34_/L98H was observed in *A. fumigatus* harbouring TR_46_/Y121F/T289A, TR_120_/F46Y/M172V/E427K and G432S and the three *A. terreus* isolates with G51A, M217I and Y491H, suggesting that acquisition of all these alterations may be an advantage in an environment where these four agents are applied. This may also be the case in *A. fumigatus* for the G54R and M220K alterations for tebuconazole and epoxiconazole, respectively.

Metconazole, prothioconazole-desthio, prochloraz and imazalil were more active against wild-type *A. fumigatus*, *A. terreus* and *A. flavus* than the other azole fungicides on a mg/L basis. The strongest reduction in susceptibility to these four agents was conferred by the TR_46_/Y121F/T289A and G54R alteration in *A. fumigatus*. Of note, TR_46_/Y121F/T289A and G54R were the only mutants with acquired resistance to prothioconazole-desthio suggesting this fungicide is not implicated in selection of TR_34_/L98H or any of the included point mutations in *A. fumigatus*. In contrast, metconazole, prochloraz and imazalil activity was reduced in TR_34_/L98H and one or both of the latter two also in G432S, TR_120_/F46Y/M172V/E427K and M220R *A. fumigatus* isolates.

The non-azole fungicides were in general not active against *Aspergillus* wild-type isolates with the exception of azoxystrobin against *A. terreus*. These agents belong to drug classes that are not used in human medicine and which, when used for plant protection are often used in combination with azole fungicides. The SDHIs bixafen and fluxapyroxad are frequently used outside Denmark in combination with azoles (prothioconazole, epoxiconazole and as of 2021 also mefentrifluconazole). Similarly, fluopyram with prothioconazole and boscalid with epoxiconazole are frequently used combinations in Denmark. Although inactive against the included wild-type *A. fumigatus* isolates, activity was observed against some of the mutant isolates of *A. fumigatus*. This was true for bixafen, boscalid and fluxapyroxad against TR_120_/F46Y/M172V/E427K (selected via the patient route), G432S (also found in an azole naïve patient), and G54A (selected via the patient route and occasionally found in the environment), and was also true for fluopyram against TR_120_/F46Y/M172V/E427K and G54A. In contrast, none of them were active against G54R, which is found in the environment in Switzerland and Thailand, nor against the two most common environmental mutants, TR_34_/L98H and TR_46_/Y121F/T289A. It remains to be understood if the use of these non-azole fungicides in combination with azoles may help prevent selection of for example G54A and G432S mutants in the environment. On the other hand, a recent study from the UK showed that in addition to azole resistance, several lineages of *A. fumigatus* carrying TR-based Cyp51A variants have also acquired resistance to three other groups of fungicides, namely methyl benzimidazole carbamate, strobiluriner (QoI) and (SDHIs) through target-site alterations in the corresponding fungicide target proteins [19]. This illustrates the capacity of *A. fumigatus* to evade a selection pressure in the environment and may explain the high level of resistance against these non-azole fungicides in the wild-type isolates included in this study.

Prothioconazole, mefentrifluconazole and the five azole metabolites did not display fungicidal activity against *A. fumigatus* or *A. flavus*, suggesting that these agents are improbable drives of resistance in these species. Once applied to the target, prothioconazole is, however, rapidly metabolised to prothioconazole-destio, which is present in the upper layers of soil, a more potent selector for resistance in plant pathogens and active against *A. fumigatus* [49,50]. Another potential caveat was that a partial inhibition pattern was observed for mefentrifluconazole against *A. fumigatus* and *A. terreus* that was not observed for the other agents and not observed against TR_34_/L98H, TR_120_/F46Y/M172V/E427K, G54A, M220K and M220R nor against *A. terreus* harbouring G51A, M217I and Y491H. Therefore, further studies are warranted before confirming that this partial inhibition of mefentrifluconazole may not present a relevant selection pressure on *Aspergillus* in the environment.

## 5. Conclusions

In conclusion, our study shows that prothioconazole, paclobutrazole, potentially mefentrifluconazole and the five well-known azole metabolites showed no or low activity against *A. fumigatus*, *A. terreus* and *A. flavus*, and thus are unlikely drivers of resistance. However, differential activity was observed for the other azole fungicides, including the prothioconazole-destio metabolite of prothioconazole, which may suggest that given the “right” circumstances, these may pose a selection pressure on all three *Aspergillus* species. Moreover, not only the recognised TR_34_/L98H and TR_46_/Y121F/T289A environmental mutations, but also seven of the eight point mutations in *A. fumigatus* and *A. terreus* increased the MIC to at least one fungicide by at least three two-fold dilutions, suggesting these mutations confer an advantage for the fungus to escape both environmental fungicides and medical azoles. Most azoles are rather persistent in soils when measured as DT_50_ (= time for disappearance of half the chemical). Half-lives are variable but range from months to years (Table 3). Field applications with azoles will expectedly impact the concentration in the upper soil layers where *A. fumigatus* can be expected to be present and in this way azole-fungicides may act as a potent selector for resistance. Despite common cases of azole resistance in plant pathogens attacking field crops [51], a recent investigation only found a few cases with resistant *A. fumigatus* in farmer fields treated with azoles, suggesting that other uses may be more important for resistance selection [19].

Taken together, these and our findings illustrate that studies focussing on identifying local practices in each country that are important for the selection of azole resistance in *Aspergillus* are of utmost importance. This is in order to identify potentially safe and beneficial practices for agricultural yield from the uses of fungicides that drive resistance in human pathogens.

## Figures and Tables

**Table 1:** In vitro activity (EUCAST MICs (mg/L) with visual complete inhibition endpoint) of 11 azole fungicides, four medical triazoles, five azole fungicide metabolites and six non azole fungicides against wild-type and *cyp51A* mutant isolates of three *Aspergillus* species.

Fungicide Class and Compound	*A. fumigatus*											*A. terreus*				*A. flavus*	
	ATCC 204305 *	Wild-Type (*n* = 7) ^a^	TR_34_/ L98H	TR_34_^(3)^/L98H	TR_46_/Y121F/T289A	TR_120_/F46Y/ M172V/ E427K	G432S	G54A	G54R	M220K	M220R	Wild-Type (*n* = 7) ^b^	G51A	M217I	Y491H	ATCC 204304 *	Wild-Type (*n* = 2) ^b^
**Triazole fungicides**																	
Prothioconazole	>32 (32->32)	>32 (4->32)	>32	>32	>32	>32	32	>32	>32	32	>32	>32 (>32)	>32	>32	>32	>32	>32
Mefentrifluconazole	>32 (16->32)	>32 (32->32)	>32	>32	>32	>32	>32	>32	>32	>32	>32	8 (1->32)	>32	>32	>32	>32	>32
Paclobutrazole	32 (16–32)	16 (16)	>32	>32	>32	>32	32	32	>32	32	>32	8 (1–32)	8	32	32	8	8; 16
Epoxiconazole	16 (8–16)	8 (4–16)	>32	>32	>32	>32	>32	16	16	>32	16	2 (1–4)	16	>32	8	16 (8–16)	8; 16
Propiconazole	8 (4–16)	8 (4–8)	>32	>32	>32	>32	>32	16	8	16	16	2 (1–8)	16	>32	8	16 (16–32)	16; 32
Tebuconazole	4 (4–8)	4 (2–4)	32	>32	>32	32	32	4	32	8	8	2 (1–8)	16	32	8	8 (4–8)	4; 8
Difenoconazole	4 (2–16)	2 (1–4)	>32	>32	>32	32	16	4	2	8	8	0.5 (0.125–2)	4	4	2	8 (4–16)	8;16
Metconazole	0.25 (0.25–0.5)	0.25 (0.25)	2	2	16	1	1	0.5	8	0.5	0.5	0.5 (0.125–1)	1	2	2	2 (2–4)	2; 4
Prothioconazole-desthio	0.125 (0.06–0.125)	0.06 (0.06–0.125)	0.125	0.25	1	0.125	0.125	0.125	4	0.125	0.125	0.5 (0.125–0.5)	0.25	0.5	0.5	0.5	0.5
**Imidazole fungicides**																	
Prochloraz	0.5	0.25 (0.25–0.5)	2	2	32	1	2	0.5	8	0.5	2	0.5 (0.25–4)	2	4	4	1	1
Imazalil	0.25 (0.25–0.5)	0.25 (0.125–0.5)	4	8	32	2	4	1	2	0.5	0.5	0.5 (0.125–1)	2	2	1	2 (1–2)	1; 2
**Medical triazoles**																	
Itraconazole	0.25 (0.25–0.5)	0.25 (0.125–0.5)	>32	>32	0.5	>32	>32	>32	>32	>32	>32	0.25 (0.06–0.5)	>32	>32	1	0.125 (0.125–0.25)	0.125; 0.25
Posaconazole	0.125 (0.06–0.125)	0.125 (0.03–0.125)	2	1	0.5	1	1	4	>32	2	0.5	0.125 (0.06–0.25)	0.5	0.5	0.5	0.125 (0.125–0.25)	0.125
Isavuconazole	1 (1–2)	1 (0.5–1)	8	16	>32	4	8	1	16	2	2	1 (0.25–4)	2	8	4	1 (1–2)	1; 2
Voriconazole	1 (0.5–2)	1 (0.25–1)	8	8	>32	2	4	1	8	2	1	0.5 (0.25–1)	2	4	4	1	1
**Fungicide metabolites**																	
1,2,4-Triazole	>16 (>16)	>16 (>16)	>16	>16	>16	>16	>16	>16	>16	>16	>16	>16 (>16)	>16	>16	>16	>16 (>16)	>16
1,2,3-Triazole	>16 (>16)	>16 (>16)	>16	>16	>16	>16	>16	>16	>16	>16	>16	>16 (>16)	>16	>16	>16	>16 (>16)	>16
Triazole alanine	>16 (>16)	>16 (>16)	>16	>16	>16	>16	>16	>16	>16	>16	>16	>16 (>16)	>16	>16	>16	>16 (>16)	>16
Triazole acetate	>16 (>16)	>16 (>16)	>16	>16	>16	>16	>16	>16	>16	>16	>16	>16 (>16)	>16	>16	>16	>16 (>16)	>16
Triazole sulfonamide	>16 (>16)	>16 (>16)	>16	>16	>16	>16	>16	>16	>16	>16	>16	>16 (>16)	>16	>16	>16	>16 (>16)	>16
**Non-azole fungicides**																	
Bixafen	>32 (>32)	>32 (>32)	>32	>32	>32	2	4	2	>32	8	>32	>32 (>32)	>32	>32	>32	>32 (>32)	>32
Boscalid	>32 (>32)	>32 (>32)	>32	>32	>32	2	2	2	>32	>32	>32	>32 (>32)	>32	>32	>32	>32 (>32)	>32
Fluxapyroxad	>32 (>32)	>32 (>32)	>32	>32	>32	2	1	2	>32	>32	>32	>32 (>32)	>32	>32	>32	>32 (>32)	>32
Fluopyram	>32 (>32)	>32 (>32)	>32	>32	>32	4	16	4	32	>32	>32	>32 (>32)	>32	>32	>32	>32 (>32)	>32
Folpet	>32 (>32)	>32 (>32)	>32	>32	>32	32	16	32	>32	32	>32	>32 (>32)	>32	32	>32	>32 (>32)	>32
Azoxystrobin	>32 (>32)	>32 (>32)	>32	>32	>32	8	>32	32	>32	>32	>32	2 (0.5–8)	>32	>32	16	>32 (>32)	>32

* MIC_50_ (range) of six repetitions; ^a^ MIC_50_ (range) of the MICs for 7 isolates; ^b^ MICs for each of the two *A. terreus* sensu stricto isolates included. The *A. terreus cyp51A* wild-type isolate with lowest MICs was found to harbour a calmodulin deletion.

**Table 2:** Relative susceptibility of mutant isolates compared to same species wild-type isolates determined as Log2(mutant MIC_50_) – Log2(wild-type MIC). Only agents with activity against wildtype isolates are included. Differences of ≥3 Log2 MICs are highlighted in bold, and of ≥4 Log2 MICs are underlined. Negative values represent cases where the mutant isolate is more susceptible than its wild-type counterpart.

	*A. fumigatus*									*A. terreus*		
	TR_34_/L98H	TR_34_^(3)^/L98H	TR_46_/Y121F/T289A	TR_120_/F46Y/M172V/E427K	G432S	G54A	G54R	M220K	M220R	G51A	M217I	Y491H
**Triazole fungicides**												
Mefentrifluconazole	0	0	0	0	0	0	0	0	0	**3**	**3**	**3**
Paclobutrazole	2	2	2	2	1	1	2	1	2	0	2	2
Epoxiconazole *	**3**	**3**	**3**	**3**	**3**	1	1	**3**	1	**3**	**5**	2
Propiconazole *	**3**	**3**	**3**	**3**	**3**	1	0	1	1	**3**	**5**	2
Tebuconazole *	**3**	**4**	**4**	**3**	**3**	0	**3**	1	1	**3**	**4**	2
Difenoconazole *	**5**	**5**	**5**	**4**	**3**	1	0	2	2	**3**	**3**	2
Metconazole	**3**	**3**	**6**	2	2	1	**5**	1	1	1	2	2
Prothioconazole-desthio	1	2	**4**	1	1	1	**6**	1	1	−1	0	0
**Imidazole fungicides**												
Prochloraz	**3**	**3**	**7**	2	**3**	1	**5**	1	**3**	2	**3**	**3**
Imazalil	**4**	**5**	**7**	**3**	**4**	2	**3**	1	1	2	2	1
**Medical triazoles**												
Itraconazole	**8**	**8**	1	**8**	**8**	**8**	**8**	**8**	**8**	**8**	**8**	2
Posaconazole	**4**	**3**	2	**3**	**3**	**5**	**9**	**4**	2	2	2	2
Isavuconazole	**3**	**4**	**6**	2	**3**	0	**4**	1	1	1	**3**	2
Voriconazole	**3**	**3**	**6**	1	2	0	**3**	1	0	2	**3**	**3**
**Strobilurin**												
Azoxystrobin	0	0	0	−3	0	−1	0	0	0	**5**	**5**	**3**

* Compounds previously associated with azole resistance selection potential [14].

**Table 3:** Comparison of azole degradation times (DT_50_ (half-life)) in soil as given in EFSA summary reports.

Azole Fungicide	DT_50_ in Soil (20 °C) (Range in Days)	Source (Link to Specific EFSA Document)
Difenoconazole	53–235	https://efsa.onlinelibrary.wiley.com/doi/epdf/10.2903/j.efsa.2011.1967
Prothioconazole	1.3–2.8	https://efsa.onlinelibrary.wiley.com/doi/epdf/10.2903/j.efsa.2007.106r
Prothioconazole-destio	7–34	https://efsa.onlinelibrary.wiley.com/doi/epdf/10.2903/j.efsa.2007.106r
Tebuconazole	20–92	https://efsa.onlinelibrary.wiley.com/doi/epdf/10.2903/j.efsa.2014.3485
Metconazole	84–598	https://www.efsa.europa.eu/en/consultations/call/180801
Propiconazole	28–131	https://efsa.onlinelibrary.wiley.com/doi/epdf/10.2903/j.efsa.2017.4887
Epoxiconazole	98–694	https://efsa.onlinelibrary.wiley.com/doi/epdf/10.2903/j.efsa.2008.138r
Imazalil	41–135	https://efsa.onlinelibrary.wiley.com/doi/epdf/10.2903/j.efsa.2010.1526
Mefentrifluconazole	104–477	https://efsa.onlinelibrary.wiley.com/doi/epdf/10.2903/j.efsa.2018.5379

## Data Availability

Not applicable.

## Supplementary Materials

The following are available online at https://www.mdpi.com/2309-608X/7/3/205/s1, Table S1: Mefentrifluconazole MIC (mg/L) with complete visual and partial spectrophotometric inhibition endpoints.

## References

[1] Lass-Flörl C., Mayr A., Aigner M., Lackner M., Orth-Höller D. (2018). A nationwide passive surveillance on fungal infections shows a low burden of azole resistance in molds and yeasts in Tyrol, Austria. Infection.

[2] Risum M., Hare R.K., Gertsen J.B., Kristensen L., Johansen H.K., Helweg-Larsen J., Abou-Chakra N., Pressler T., Skov M., Jensen-Fangel S. (2020). Azole-Resistant Aspergillus fumigatus Among Danish Cystic Fibrosis Patients: Increasing Prevalence and Dominance of TR34/L98H. Front. Microbiol..

[3] Cho S.Y., Lee D.G., Choi J.K., Lee H.J., Kim S.H., Park S.H., Choi S.M., Choi J.H., Yoo J.H., Park Y.J. (2017). Characteristics of culture-positive invasive pulmonary aspergillosis in patients with hematologic diseases: Comparison between Aspergillus fumigatus and non-fumigatus Aspergillus species. Medicine.

[4] Klich M.A. (2007). Aspergillus flavus: The major producer of aflatoxin. Mol. Plant Pathol..

[5] Samson R.A., Peterson S.W., Frisvad J.C., Varga J. (2011). New species in Aspergillus section Terrei. Stud. Mycol..

[6] Lass-Flörl C., Rath P.-M., Niederwieser D., Kofler G., Würzner R., Krezy A., Dierich M.P. (2000). Aspergillus terreus infections in haematological malignancies: Molecular epidemiology suggests association with in-hospital plants. J. Hosp. Infect..

[7] Kozakiewicz Z. (1989). Aspergillus species on stored products. Mycol. Pap..

[8] Leger R.J.S., Screen S.E., Shams-Pirzadeh B. (2000). Lack of Host Specialization inAspergillus flavus. Appl. Environ. Microbiol..

[9] Ullmann A.J., Aguado J.M., Arikan-Akdagli S., Denning D.W., Groll A.H., Lagrou K., Lass-Flörl C., Lewis R.E., Munoz P., Verweij P.E. (2018). Diagnosis and management of Aspergillus diseases: Executive summary of the 2017 ESCMID-ECMM-ERS guideline. Clin. Microbiol. Infect..

[10] Denning D.W.D., Cadranel J., Beigelman-Aubry C., Ader F., Chakrabarti A., Blot S., Ullmann A.J., Dimopoulos G., Lange C., Ullman A. (2016). Chronic pulmonary aspergillosis: Rationale and clinical guidelines for diagnosis and management. Eur. Respir. J..

[11] Wilson D.T., Dimondi V.P., Johnson S.W., Jones T.M., Drew R.H. (2016). Role of isavuconazole in the treatment of invasive fungal infections. Ther. Clin. Risk Manag..

[12] Lestrade P.P., Bentvelsen R.G., Schauwvlieghe A.F.A.D., Schalekamp S., van der Velden W.J.F.M., Kuiper E.J., van Paassen J., van der Hoven B., van der Lee H.A., Melchers W.J.G. (2019). Voriconazole Resistance and Mortality in Invasive Aspergillosis: A Multicenter Retrospective Cohort Study. Clin. Infect. Dis..

[13] Lestrade P.P.A., Meis J.F., Melchers W.J.G., Verweij P.E. (2019). Triazole resistance in Aspergillus fumigatus: Recent insights and challenges for patient management. Clin. Microbiol. Infect..

[14] Meis J.F., Chowdhary A., Rhodes J.L., Fisher M.C., Verweij P.E. (2016). Clinical implications of globally emerging azole resistance in Aspergillus fumigatus. Philos. Trans. R. Soc. B Biol. Sci..

[15] Snelders E., Camps S.M.T., Karawajczyk A., Schaftenaar G., Kema G.H.J., van der Lee H.A., Klaassen C.H., Melchers W.J.G., Verweij P.E. (2012). Triazole fungicides can induce cross-resistance to medical triazoles in Aspergillus fumigatus. PLoS ONE.

[16] Mortensen K.L., Jensen R.H., Johansen H.K., Skov M., Pressler T., Howard S.J., Leatherbarrow H., Mellado E., Arendrup M.C. (2011). Aspergillus species and other molds in respiratory samples from patients with cystic fibrosis: A laboratory-based study with focus on Aspergillus fumigatus azole resistance. J. Clin. Microbiol..

[17] Buil J.B., Hare R.K., Zwaan B.J., Arendrup M.C., Melchers W.J.G., Verweij P.E. (2019). The fading boundaries between patient and environmental routes of triazole resistance selection in Aspergillus fumigatus. PLoS Pathog..

[18] Stensvold C.R., Jørgensen L.N., Arendrup M.C. (2012). Azole-Resistant Invasive Aspergillosis: Relationship to Agriculture. Curr. Fungal Infect. Rep..

[19] Gisi U. (2014). Assessment of selection and resistance risk for demethylation inhibitor fungicides in Aspergillus fumigatus in agriculture and medicine: A critical review. Pest Manag. Sci..

[20] Fraaije B., Atkins S., Hanley S., Macdonald A., Lucas J. (2020). The Multi-Fungicide Resistance Status of Aspergillus fumigatus Populations in Arable Soils and the Wider European Environment. Front. Microbiol..

[21] Vermeulen E., Lagrou K., Verweij P.E. (2013). Azole resistance in Aspergillus fumigatus: A growing public health concern. Curr. Opin. Infect. Dis..

[22] Zhang J., Lopez Jimenez L., Snelders E., Debets A.J.M., Rietveld A.G., Zwaan B.J., Verweij P.E., Schoustra S.E. (2021). Dynamics of Aspergillus fumigatus in azole-fungicide-containing plant waste, the Netherlands, 2016–2017. Appl. Environ. Microbiol..

[23] Verweij P.E., Lucas J.A., Arendrup M.C., Bowyer P., Brinkmann A.J.F., Denning D.W., Dyer P.S., Fisher M.C., Geenen P.L., Gisi U. (2020). The one health problem of azole resistance in Aspergillus fumigatus: Current insights and future research agenda. Fungal Biol. Rev..

[24] Bueid A., Howard S.J., Moore C.B., Richardson M.D., Harrison E., Bowyer P., Denning D.W. (2010). Azole antifungal resistance in Aspergillus fumigatus: 2008 and 2009. J. Antimicrob. Chemother..

[25] Rivero-Menendez O., Soto-Debran J.C., Medina N., Lucio J., Mellado E., Alastruey-Izquierdo A. (2019). Molecular identification, antifungal susceptibility testing, and mechanisms of azole resistance in Aspergillus species received within a surveillance program on antifungal resistance in Spain. Antimicrob. Agents Chemother..

[26] Hermida-Alava K., Brito Devoto T., Sautua F., Gordó M., Scandiani M., Formento N., Luque A., Carmona M., Cuestas M.L. (2021). Antifungal susceptibility profile and molecular identification of Cyp51C mutations in clinical and environmental isolates of Aspergillus flavus from Argentina. Mycoses.

[27] Sharma C., Kumar R., Kumar N., Masih A., Gupta D., Chowdhary A. (2018). Investigation of Multiple Resistance Mechanisms in Voriconazole-Resistant Aspergillus flavus Clinical Isolates from a Chest Hospital Surveillance in Delhi, India. Antimicrob. Agents Chemother..

[28] Paul R.A., Rudramurthy S.M., Meis J.F., Mouton J.W., Chakrabarti A. (2015). A Novel Y319H Substitution in CYP51C Associated with Azole Resistance in Aspergillus flavus. Antimicrob. Agents Chemother..

[29] Lucio J., Gonzalez-Jimenez I., Rivero-Menendez O., Alastruey-Izquierdo A., Pelaez T., Alcazar-Fuoli L., Mellado E. (2020). Point Mutations in the 14-α Sterol Demethylase Cyp51A or Cyp51C Could Contribute to Azole Resistance in Aspergillus flavus. Genes.

[30] Paul R.A., Rudramurthy S.M., Dhaliwal M., Singh P., Ghosh A.K., Kaur H., Varma S., Agarwal R., Chakrabarti A. (2018). Voriconazole resistance in clinical and environmental isolates of Aspergillus flavus—frequency and investigation into the role of multidrug efflux pumps. Antimicrob. Agents Chemother..

[31] Ukai Y., Kuroiwa M., Kurihara N., Naruse H., Homma T., Maki H., Naito A. (2018). Contributions of yap1 Mutation and Subsequent atrF Upregulation to Voriconazole Resistance in Aspergillus flavus. Antimicrob. Agents Chemother..

[32] Duong T.M.N., Nguyen P.T., Van Le T., Nguyen H.L.P., Nguyen B.N.T., Nguyen B.P.T., Nguyen T.A., Chen S.C.A., Barrs V.R., Halliday C.L. (2020). Drug-resistant aspergillus flavus is highly prevalent in the environment of Vietnam: A new challenge for the management of aspergillosis?. J. Fungi.

[33] Hong S.-B., Go S.-J., Shin H.-D., Frisvad J.C., Samson R.A. (2005). Polyphasic taxonomy of Aspergillus fumigatus and related species. Mycologia.

[34] Arendrup M.C., Jensen R.H., Grif K., Skov M., Pressler T., Johansen H.K., Lass-Flörl C. (2012). In vivo emergence of aspergillus terreus with reduced azole susceptibility and a Cyp51a M217I Alteration. J. Infect. Dis..

[35] Arendrup M.C., Meletiadis J., Mouton J.W., Guinea J., Cuenca-Estrella M., Lagrou K., Howard S.J. (2016). EUCAST technical note on isavuconazole breakpoints for Aspergillus, itraconazole breakpoints for Candida and updates for the antifungal susceptibility testing method documents. Clin. Microbiol. Infect..

[36] Zhang J., Snelders E., Zwaan B.J., Schoustra S.E., Meis J.F., van Dijk K., Hagen F., van der Beek M.T., Kampinga G.A., Zoll J. (2017). A Novel Environmental Azole Resistance Mutation in Aspergillus fumigatus and a Possible Role of Sexual Reproduction in Its Emergence. MBio.

[37] Alvarez-Moreno C., Lavergne R.-A., Hagen F., Morio F., Meis J.F., Le Pape P. (2017). Azole-resistant Aspergillus fumigatus harboring TR34/L98H, TR46/Y121F/T289A and TR53 mutations related to flower fields in Colombia. Sci. Rep..

[38] Hare R.K., Gertsen J.B., Astvad K.M.T., Degn K.B., Løkke A., Stegger M., Andersen P.S., Kristensen L., Arendrup M.C. (2019). In Vivo Selection of a Unique Tandem Repeat Mediated Azole Resistance Mechanism (TR120) in Aspergillus fumigatus cyp51A, Denmark. Emerg. Infect. Dis..

[39] Bader O., Tünnermann J., Dudakova A., Tangwattanachuleeporn M., Weig M., Groß U., Hoberg N., Geibel S., Vogel E., Büntzel J. (2015). Environmental isolates of azole-resistant Aspergillus fumigatus in Germany. Antimicrob. Agents Chemother..

[40] Prigitano A., Esposto M.C., Romanò L., Auxilia F., Tortorano A.M. (2019). Azole-resistant Aspergillus fumigatus in the Italian environment. J. Glob. Antimicrob. Resist..

[41] Sharma C., Nelson-Sathi S., Singh A., Radhakrishna Pillai M., Chowdhary A. (2019). Genomic perspective of triazole resistance in clinical and environmental Aspergillus fumigatus isolates without cyp51A mutations. Fungal Genet. Biol..

[42] Sharma C., Hagen F., Moroti R., Meis J.F., Chowdhary A. (2015). Triazole-resistant Aspergillus fumigatus harbouring G54 mutation: Is it de novo or environmentally acquired?. J. Glob. Antimicrob. Resist..

[43] Leonardelli F., Theill L., Nardin M.E., Macedo D., Dudiuk C., Mendez E., Gamarra S., Garcia-Effron G. (2017). First itraconazole resistant Aspergillus fumigatus clinical isolate harbouring a G54E substitution in Cyp51Ap in South America. Rev. Iberoam. Micol..

[44] Riat A., Plojoux J., Gindro K., Schrenzel J., Sanglard D. (2018). Azole Resistance of Environmental and Clinical Aspergillus fumigatus Isolates from Switzerland. Antimicrob. Agents Chemother..

[45] Tangwattanachuleeporn M., Minarin N., Saichan S., Sermsri P., Mitkornburee R., Groß U., Chindamporn A., Bader O. (2017). Prevalence of azole-resistant Aspergillus fumigatus in the environment of Thailand. Med. Mycol..

[46] Jeanvoine A., Rocchi S., Reboux G., Crini N., Crini G., Millon L. (2017). Azole-resistant Aspergillus fumigatus in sawmills of Eastern France. J. Appl. Microbiol..

[47] Dauchy C., Bautin N., Nseir S., Reboux G., Wintjens R., Le Rouzic O., Sendid B., Viscogliosi E., Le Pape P., Arendrup M.C. (2018). Emergence of Aspergillus fumigatus azole resistance in azole-naïve patients with chronic obstructive pulmonary disease and their homes. Indoor Air.

[48] Cao D., Wu R., Dong S., Wang F., Ju C., Yu S., Xu S., Fang H., Yu Y. (2020). Five-Year Survey (2014 to 2018) of Azole Resistance in Environmental Aspergillus fumigatus Isolates from China. Antimicrob. Agents Chemother..

[49] Parker J.E., Warrilow A.G.S., Cools H.J., Martel C.M., Nes W.D., Fraaije B.A., Lucas J.A., Kelly D.E., Kelly S.L. (2011). Mechanism of binding of prothioconazole to Mycosphaerella graminicola CYP51 differs from that of other azole antifungals. Appl. Environ. Microbiol..

[50] EFSA (2007). Conclusion on the Peer Review of Prothioconazole.

[51] Lucas J.A., Hawkins N.J., Fraaije B.A., Sariaslani S., Gadd G.M. (2015). The Evolution of Fungicide Resistance. Advances in Applied Microbiology.

