# Supplementary material for: Dissection of the Activity of Agricultural Fungicides against Clinical Aspergillus Isolates with and without Environmentally and Medically Induced Azole Resistance
Source: J Fungi (Basel). 2021 Mar 11;7(3):205. doi: 10.3390/jof7030205 (PMC8001900; doi:10.3390/jof7030205)
Supplement: Supplementary file 1 [file jof-07-00205-s001.pdf]

Table S1. Mefentrifluconazole MIC (mg/L) with complete visual and partial spectrophotometric inhibition endpoints.

Mefentrifluconazole	*A. fumigatus*										*A. terreus*				*A. flavus*
	wt (n=7)[a]	TR$_{34}$/L98H	TR$_{34}^{(3)}$/L98H	TR$_{46}$/Y121F/T289A	TR$_{120}$/F46Y/M172V/E427K	G432S	G54A	G54R	M220K	M220R	wt (n=7)[a]	G51A	M217I	Y491H	wt (n=2)[b]
Complete visual inhib.	>16 (>16)	>16	>16	>16	>16	>16	>16	8/>16	>16	>16	8 (1->32)	>16	>16	>16	>16
Spec-90% inh.	>16 (8->16)	>16	>16	>16	>16	>16	>16	8	>16	>16	8 (1-32)	>16	>16	>16	>16
Spec-80% inh.	8 (8->16)	>16	>16	>16	>16	>16	>16	8	>16	>16	4 (0.5-16)	>16	>16	>16	>16
Spec-70% inh.	8 (8->16)	>16	>16	>16	>16	16	>16	4	>16	>16	4 (0.25-16)	>16	>16	16	>16
Spec-60% inh.	8 (2-8)	>16	>16	>16	>16	8	>16	4	>16	>16	2 (0.25-16)	16	>16	16	>16
Spec-50% inh.	4 (2-8)	>16	>16	>16	>16	8	16	4	16	>16	2 (0.25-16)	16	>16	16	>16

[a] MIC_{50} (range) of the MICs for 7 isolates.
